# Post-tuberculosis treatment paradoxical reactions

**DOI:** 10.1007/s15010-024-02310-0

**Published:** 2024-07-02

**Authors:** Sabine M. Hermans, Onno W. Akkerman, Graeme Meintjes, Martin P. Grobusch

**Affiliations:** 1 Centre for Tropical Medicine and Travel Medicine, Department of Infectious Diseases, Amsterdam Public Health-Global Health, Amsterdam Infection and Immunity, Amsterdam UMC, Location University of Amsterdam, Amsterdam, The Netherlands; 2 Department of Global Health, Amsterdam Institute for Global Health and Development, Amsterdam UMC, Location University of Amsterdam, Amsterdam, The Netherlands; 3 Department of Pulmonary Diseases and Tuberculosis, University Medical Centre Groningen, University of Groningen, Groningen, The Netherlands; 4 University Medical Centre Groningen, TB Centre Beatrixoord, University of Groningen, Groningen, The Netherlands; 5 Institute of Infectious Disease and Molecular Medicine, University of Cape Town, Cape Town, South Africa; 6 Department of Medicine, University of Cape Town, Cape Town, South Africa; 7 Blizard Institute, Faculty of Medicine and Dentistry, Queen Mary University of London, London, UK; 8 Institute of Tropical Medicine, University of Tuebingen, Tübingen, Germany; 9 Centre de Recherches Médicales en Lambaréné (CERMEL), Lambaréné, Gabon; 10 Masanga Medical Research Unit (MMRU), Masanga, Sierra Leone

**Keywords:** Tuberculosis, Recurrence, Lymph node, Paradoxical reaction, Paradoxical upgrading, Paradoxical response

## Abstract

Paradoxical reactions (PR) to tuberculosis (TB) treatment are common during treatment, but have also been described after treatment. A presentation with recurrent signs or symptoms of TB after cure or completion of prior treatment needs to be differentiated between microbiological relapse and a paradoxical reaction. We searched all published literature on post-treatment PR, and present a synthesis of 30 studies, focusing on the epidemiology, diagnosis and management of this phenomenon. We report an additional case vignette. The majority of studies were of lymph node TB (LN-TB), followed by central nervous system TB (CNS-TB). A total of 112 confirmed and 42 possible post-treatment PR cases were reported. The incidence ranged between 3 and 14% in LN-TB and was more frequent than relapses, and between 0 and 2% in all TB. We found four reports of pulmonary or pleural TB post-treatment PR cases. The incidence did not differ by length of treatment, but was associated with younger age at initial diagnosis, and having had a PR (later) during treatment. Post-treatment PR developed mainly within the first 6 months after the end of TB treatment but has been reported many years later (longest report 10 years). The mainstays of diagnosis and management are negative mycobacterial cultures and anti-inflammatory treatment, respectively. Due to the favourable prognosis in LN-TB recurrent symptoms, a short period of observation is warranted to assess for spontaneous regression. In CNS-TB with recurrent symptoms, immediate investigation and anti-inflammatory treatment with the possibility of TB retreatment should be undertaken.

**Supplementary Information:**

The online version contains supplementary material available at 10.1007/s15010-024-02310-0.

## Introduction

In non-tuberculosis (TB)-endemic settings, the presentation of a patient with recurrent signs or symptoms of TB after cure or completion of prior treatment often leads to dilemmas regarding differentiating microbiological relapse from paradoxical reactions (PR). In recurrent pulmonary symptoms, complications like bacterial superinfection of residual cavities or aspergillosis must also be considered in the differential diagnosis. The pathogenesis of PRs is related to an immune-driven inflammatory reaction to residual *Mycobacterium tuberculosis* antigen in tissues rather than representing treatment failure or microbiological relapse. In TB endemic settings, reinfection might also be a consideration, although it is unlikely that reinfection would present with recurrence of the same signs or symptoms as the initial presentation for extrapulmonary manifestations.

Paradoxical reactions are defined by clinical or radiological worsening of pre-existing tuberculous lesions, or the development of new lesions, in patients receiving anti-tuberculous medication who initially improved on treatment [1]. These reactions are often associated with the initiation of antiretroviral therapy in patients with HIV, where they are called paradoxical TB-IRIS. The incidence of PRs during treatment differs in different populations, and is estimated at 18% in HIV-positive patients after starting ART (with some estimates in high risk groups as high as 50%), and between 10 and 25% in HIV-negative patients [2–5]. The incidence among HIV-negative patients also differs by disease site, with PRs occurring much more frequently in extrapulmonary TB (25%) than pulmonary TB (2%) [6]. Some localizations of extrapulmonary TB are associated with more PR than others, in particular cervical lymph node TB and central nervous system TB [4, 7]. However, this might be due to ascertainment bias, as PR in those sites is more likely to lead to symptoms and be recognized.

The timing of these reactions can be divided into early and late, with two-thirds of PRs presenting early (within 1–4 months of treatment initiation) [8]. The timing seems to differ by the site involved: the median time in cohorts that include all forms of TB is shorter than in those only including EPTB [7]. There are suggestions that the timing of PR in CNS TB is later than in other sites of TB, which might be due to differences in drug penetration or to dynamics of immunological processes because of the blood–brain barrier [6].

Paradoxical reactions have also been described after the end of TB treatment [9], in which situation it is important but difficult to differentiate from microbiologic relapse. Altogether, post-treatment PR is an under-investigated area of TB medicine and will be the topic of this review. We searched all published literature on post-treatment PR, and present a synthesis thereof, focusing on the epidemiology, diagnosis and management of this phenomenon.

## Literature search

We performed a literature review in PubMed using the search terms ‘tuberculosis’, ‘recurrence’ and ‘paradoxical reaction’ plus synonyms of all literature up to 1 March 2024 (for search terms see Table S1, Appendix). We also screened the reference lists of included publications and of reviews that were identified during our search. We included all publications which reported primary data on paradoxical reactions after the end of TB treatment (defined as a minimum of 1 month after completion of treatment), irrespective of study design. This was done to ensure that we did not include patients with PRs that had developed during treatment; we therefore excluded reports on residual lymph nodes or paradoxical reactions occurring at the end of treatment. We excluded case reports that were obvious relapses, but included all case reports describing recurrences which were considered by the authors to be a PR, and we categorized them as possible or confirmed PRs (for definitions see below).

For all the cases of PR after the end of treatment, we extracted data on timing after the end of treatment (in months), baseline and clinical characteristics, diagnosis, management and outcome, if these were reported. We categorised all recurrences after treatment as relapses (defined as culture-positive), confirmed PR (defined as culture negative OR culture not done with spontaneous regression), or possible PR (defined as [culture not done OR culture negative] AND retreated, irrespective of supposed treatment effect). For each study with an appropriate design (cohort or trial), we calculated the cumulative incidence of post-treatment PR, by dividing the number of confirmed PR by the number of participants that had completed study-specific follow-up. Search, screening and data extraction were done by one person (SH), in cases of doubt another team member was consulted (MPG).

**Fig. 1:**
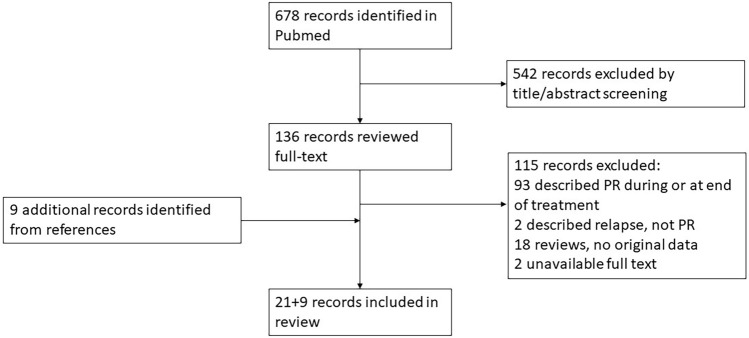
Flow diagram of literature screening and inclusion

We identified 678 articles for further screening and included 21 of those in this review; we identified nine additional publications from reference lists (see the screening flow chart in Fig. 1). Two case reports were in Japanese, but we were able to extract some data from the English abstract [10, 11].

The 30 studies included were rather diverse. Four were randomised controlled trials, one of which consisted of two publications [12–16]; ten were cohort studies (both retrospective and prospective) [4, 7–9, 17–22], of which three reported on increasing sizes of the same cohort ([8, 9, 20] and 16 were case descriptions (case reports or case series) [10, 11, 23–35]. The studies were published between 1979 and 2023, and originated from Asia [14], Europe [13], North America [2] and Africa [2]. The included studies are described and summarized in Table 1 (cohorts and RCTs) and Table 2 (case reports).

**Table 1:** Description and results of the trials and cohort studies included, by TB type

Year	First author	Country of study	Study design	Study population	HIV status	N (complete FU)	FU time (total)	New events post-Rx^a^	Incidence of post-Rx PR (%)
1979	Campbell	UK	RCT: S2HR18 vs S2HE18	LN TB	NR	90	36 M	6 (0/0/6)	7
1985	BTS^b,d^	UK	RCT: E2RH9 vs E2RH18	LN TB	NR	113	36 M	12 (0/4/8)	7
1988	BTS^**d**^	UK	RCT: E2RH9 vs E2RH18	LN TB	NR	73	60 M	13 (0/4/9)	12
1993	Campbell	UK	RCT: E2RH9, Z2RH9 or Z2RH6	LN TB	NR	165	30 M	23 (0/9/14)	8
2005	Polesky	US	Prospective cohort	LN TB	5%	94	Mean 10 M after end of Rx	8 (1/0/7)	7
2005	Hawkey	UK	Retrospective cohort	LN TB	Negative	109	1 year after Rx	3 (0/0/3)	3
2010	Park^e^	South Korea	Prospective cohort	LN TB	negative	75	NR	9 (0/0/8)	11
2013	Park^e^	South Korea	Prospective cohort	LN TB	Negative	154	Median 24M	24 (0/2/22)	14
2015	Yu^e^	South Korea	Retrospective and prospective cohort	LN TB	Negative	467	NR	34 (0/16/18)	4
2019	Seok	South Korea	Prospective cohort	Cervical LN TB	Negative	165	NR	5% (NR/2/NR)	
1984	BTS^b^	UK	RCT: SHRZ6 vs EHRZ6 vs EHR9	PTB	NR	373	36M	9 (6/2/0)^c^	0
2002	Choi	South Korea	Retrospective cohort	Pleural effusion TB	Negative	141	12-27 (mean 19M)	1 (0/1/0)	0
2013	Geri	France	Retrospective cohort	EPTB, hospitalized, 72% LN TB	Negative	76	NR	NR (min 2) (0/0/2)	3
2012	Worodria	Uganda	Prospective cohort	All TB, starting ART	Positive	254	1 year after ART initiation?	5 (0/0/5)	2
2016	Brown	UK	Cohort study and nested case control	All TB	7%	1817	1 visit after Rx	NR (min 1) (0/0/1)	0

*ART* antiretroviral therapy, *BTS* British Thoracic Society Research Committee, *E* ethambutol, *FU* follow-up, *H* isoniazid, *LN* lymph node, *post-Rx* after end of TB treatment, *PR* paradoxical reaction, *PTB* pulmonary tuberculosis, *min* minimum, *M* months, *NR* not reported, *RCT* randomised controlled trial, *R* rifampicin, *Rx* treatment, *S* streptomycin, *TB* tuberculosis, *UK* United Kingdom, *US* United States, *Z* pyrazinamide

^a^Total number of events after end of treatment during follow-up (relapse/possible PR/confirmed PR)

**Fig. 2:**
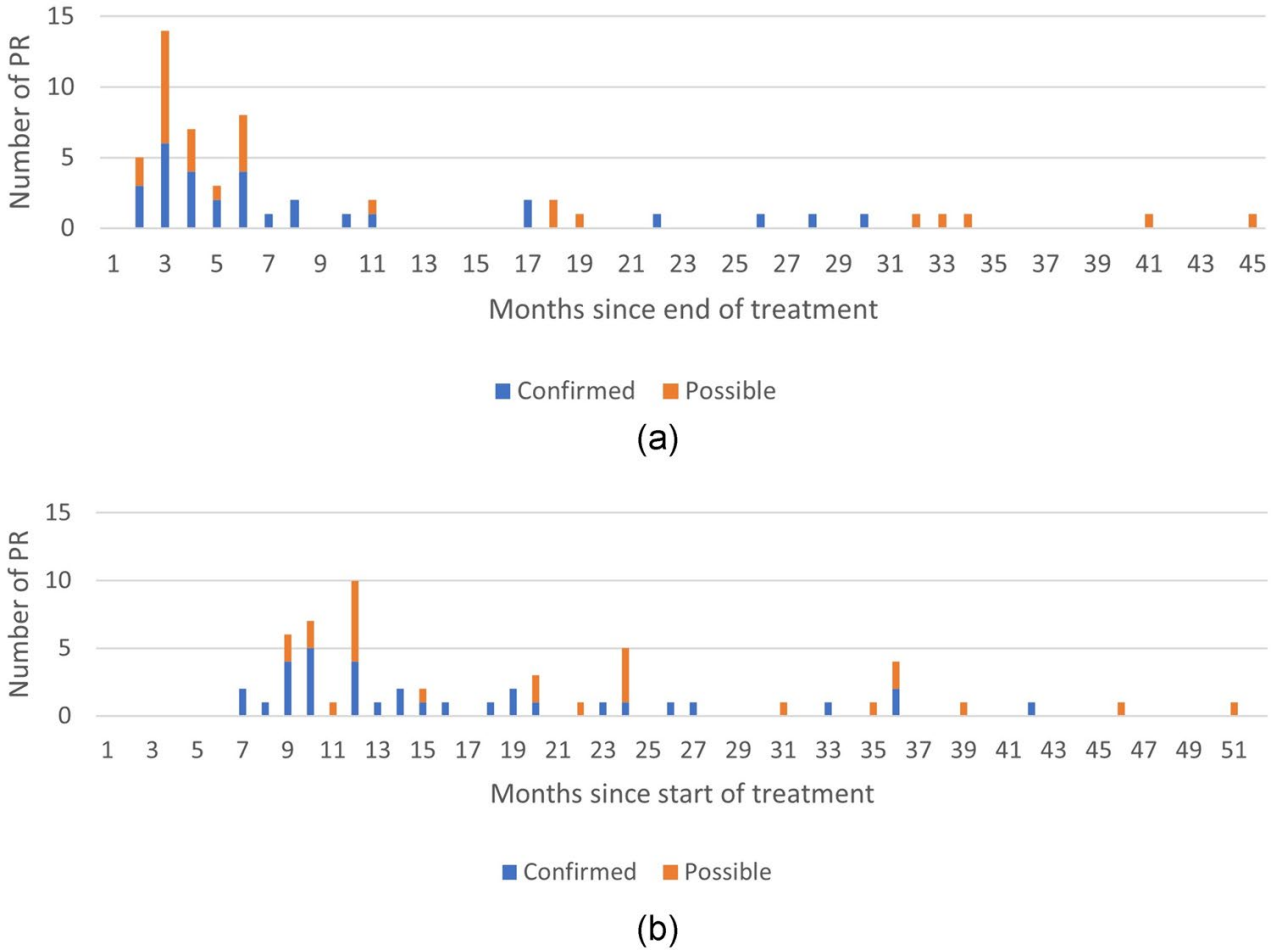
Timing of post-treatment PR, stratified by category; **a** months since the end of TB treatment and **b** months after the start of TB treatment. Four recurrences in the first month after treatment are included in the second graph, but not in the first; as the primary data reported was insufficient to exclude them from the first [20]. The recurrences from the 1985 BTS publication were all included as possible PRs as the data stratified by month did not provide information on culture results [14]. As per the exclusion criteria, we excluded cases of PR that occurred at the end of treatment or in the first month after treatment. The data underlying this Figure are included in Table S3 of the appendix. An outlier (a confirmed PR) was reported at 132 months or 11 years (**a**) and 120 months or 10 years (**b**), respectively. Our case vignette was a confirmed case reported at 100 months (8.3 years) and 106 months (8.9 years), respectively

^b^Contributed to the cases reported in Fig. 2

^c^1 event was neither relapse or PR, but bacterial infection

^d^Reports of the same trial but with longer follow-up time

^e^Reports of the same cohort over time (with different sample sizes)

**Table 2:** Details of included case reports

Year	First author	Country	Age	Sex	Type of TB	HIV status	Timing^a^	Location of PR	Culture	Management	PR du- ring Rx	Category	Recurrent PR
1994	Carter	US	25	Female	PTB	Negative	22	LN	Negative	No further treatment, resolved	Yes	Confirmed	No
2003	Ramos	Spain	33	Male	Disseminated	Positive	3	LN	Negative	None, cervical LN appear and disappear spontaneously	Yes	Confirmed	Yes
2004	Mert	Turkey	48	Female	LN TB	NR	4	LN	Negative	Surgical removal, no treatment, FU for 8 years fine	Yes	Possible	No
2004	Takeshima	Japan	26	Male	Tuberculoma	Negative	4	Meningitis	Not done	Retreated for 12M with good response	Yes	Confirmed	No
2006	Kondo	Japan	NR	NR	NR	NR	3	NR	NR	Prednisolone spontaneous resolution	No	Confirmed	No
2007	Huyst	Belgium	35	Female	Disseminated	Positive	34	LN	Negative	Retreatment for 8M with good response	Yes	Confirmed	No
2010	Takao	Japan	NR	NR	PTB	NR	6	Pleural tuberculoma	Not done	Spontaneous resolution within 12 M	No	Possible	No
2012	Lee	UK	78	Female	Abdominal TB	NR	19	Intestinal perforation	NR	Small bowel resection; post-operative MI, died	No	Possible	No
2012	Malhotra	India	32	Female	TBM	Negative	4	Arachnoiditis	NR	Not reported	No	Possible	No
2012	Shah	India	2.5	Male	TBM	NR	2	Tuberculoma	NR	Retreated 12 M, had recurrence of seizures after 6 M: suggestive TBM, retreated with MDR Rx, LFU	NR	Possible	Yes
2012	Shah	India	4	Male	TBM	NR	11	Tuberculomas	NR	Retreated for 18 M, including steroids, LFU	Yes	Confirmed	No
2013	Yalcinsoy	Turkey	NR	NR	PTB and LN TB	Negative	8	LN	NR	Regressed without treatment	No	Confirmed	No
2014	Krishnaraj	India	41	Male	PTB	Positive	6	Meningitis	Not done	Retreated with 8M, recovery. 2nd PR after 6M (abscesses), CD4 992, ZN positive no culture; 'anti-edema measures' and recovered	No	Confirmed	Yes
2018	Machida	Japan	27	Female	TBM	Negative	120	Tuberculomas	Negative	3 M prednison 30 mg/day, tuberculoma disappeared, tapered off over 1 year. No recurrence 12M later	Yes	Confirmed	No
2023	Prasai	Nepal	18	Female	Intestinal TB	NR	6	Cecal perforation	NR	Hemicolectomy, no additional TB-Rx or steroids	No	Confirmed	No
2023	Armange^b^	France	26	Female	PTB	Negative	7	LN and presternal mass	Negative	(1) laparotomy, no treatment, (2) and (3) no treatment, spontaneous resolution and (4) patient wish retreatment and infliximab, no change in symptoms (1 year FU)	Yes	Possible	Yes

*FU* follow-up, *LFU* loss to follow-up, *LN* lymph node, *post-Rx* after end of TB treatment, *PR* paradoxical reaction, *PTB* pulmonary tuberculosis, *min* minimum, *M* months, *MDR* multi-drug resistant, *MI* myocardial infarction, *NR* not reported, *Rx* treatment, *TB* tuberculosis, *UK* United Kingdom, *US* United States, *ZN* Ziehl Neelsen

^a^Since end of treatment (months; first post-treatment PR)

^b^Four episodes of post-treatment PR: approx. 1 M (17 days after delivery), 7 M, 3 years and 4.5 years post-treatment

## Reported incidence of post-treatment PR

The 15 studies that had designs allowing determination of incidence are included in Table 1. The majority of the studies (10/15) were among patients with lymph node TB, and the study-specific incidence of post-treatment PR in that population ranged between 3 and 14% for confirmed PR, and 3–18% for possible PR, respectively, during follow-up times ranging from 10 months up to 36 months after treatment completion. Studies in a population of all TB patients found low incidence (0–2%) [7, 21], and no pulmonary PR. However, diagnosing pulmonary PR post-treatment is very challenging because in addition to relapse, many clinicians will consider or treat for bacterial infections, especially if there is bronchiectasis. Thus, pulmonary PR may be truly rare or uncommon because it is not ascertained for these reasons. There were no cohort studies of CNS TB reporting on these outcomes. There was only one cohort of HIV positive TB patients, which also showed a low incidence of 2%; however, the follow-up time was short [21].

Looking specifically at the data from the trials, there were no differences in post-treatment PR incidence by different treatments lengths: 6–9% with 18 months, 9–10% with nine months and 7% with 6 months treatment duration, respectively (Table S1).

All studies reported much higher numbers of PR than relapses (Table 1). The studies of Park et al., Park et al. and Yu et al. [8, 9, 20] describe an expanding cohort of LN TB patients from the same university clinic in South Korea, with the latter publication largely adding patients treated longer time ago (1997–2007). There was a much higher proportion of retreatment of post-treatment events despite cultures remaining negative in the publication spanning the period 1997–2012 than in the period 2008–2012 (Table 1). Yu et al. state that this change in management was a result of their increased confidence in spontaneous regression seeing their increasing expertise in this area [8].

Polesky et al. reported a similar proportion of the post-treatment PR occurring in the same location of the original disease, and in a new site [18]. Park et al. reported 71% enlargement of nodes at previous sites, 21% new nodes and 13% sinus formation [20]. Yu et al. observed new nodes after completing TB treatment more frequently in their late PR group compared to the early PR group [8]. Both reported sinus formation being more common in early PR, or PR during treatment, compared to late PR [8, 9].

## Case reports

See Table 2 for summary descriptions of the 15 case reports included. Table 3 includes the details of the 11 case descriptions that could be extracted from the cohort studies [7, 13, 17, 21, 22].

**Table 3:** Details of post-treatment PR cases extracted from trials and cohort studies

First author	Year	Age	Sex	Type of TB	HIV status	Timing^a^	Location of PR	Culture	Management	PR during Rx	Category	Recurrent PR
Choi	2002	29	Male	TB pleural effusion	Negative	3	Pleural tuberculoma	Not done	Retreated, symptoms resolved	NR	Possible	No
Worodria	2012	44	Male	PTB, 6M Rx. baseline CD4 5	Positive, CD4 95 at PR	3	Ankle	Not done	Aspiration and NSAIDs with good response; recurrence after 1 year (same location/symptoms), management identical	Yes	Confirmed	Yes
Worodria	2012	40	Male	PTB, 8M Rx. baseline CD4 52. IRIS with generalised LN after 4M of ART	Positive, CD4 232 3 M prior to PR	11	LN	Not done	Repeated aspiration 3× in 5 M, antibiotics (ampicillin + cloxacillin), NSAIDS.	No	Confirmed	No
Worodria	2012	27	Female	PTB, 6M Rx. baseline CD4 4. IRIS at 2 weeks of ART	Positive, CD4 151 2 M prior to PR	3	LN	Not done	NSAIDs	Yes	Confirmed	No
Worodria	2012	45	Female	PTB, baseline CD4 21	Positive, CD4 at PR 222	5	PTB and EPTB	NR	NR	NR	Possible	No
Worodria	2012	36	Male	PTB, baseline CD4 27	Positive, CD4 at PR 257	4	EPTB	NR	NR	NR	Possible	No
Brown	2016	NR	NR	NR	NR	35	NR	NR	NR	NR	Possible	NR
Seok	2019	NR	NR	LN TB	Negative	2	NR	NR	NR	NR	Possible	No
Seok	2019	NR	NR	LN TB	Negative	32	NR	NR	NR	NR	Possible	No

*ART* antiretroviral therapy, *EPTB* extrapulmonary tuberculosis, *IRIS* immune reconstitution inflammatory syndrome, *LN* lymph node, *PR* paradoxical reaction, *PTB* pulmonary tuberculosis, *M* months, *NR* not reported, *NSAIDs* non-steroidal anti-inflammatory drugs, *TB* tuberculosis

^a^Since end of treatment (months; first post-treatment PR)

Patients whose original disease location was pulmonary or disseminated TB presented with post-treatment PR in different sites to the original presentation, being LN and the central nervous system (CNS), whereas patients presenting with their TB in the CNS or in the abdomen developed their PR in the same disease location. There were four descriptions of pulmonary locations of post-treatment PR, of which two were pleural tuberculomas [10, 13, 17, 21]. Two of the five patients with a LN-TB post-treatment PR reported suppuration and draining sinus formation [30, 31], one of whom was HIV positive [31].

Six cases had multiple episodes of PR after the end of treatment. This occurred among three of the seven HIV positive cases, who had several recurrent PR episodes while their CD4 count was increasing [21, 24, 31]. This was also the case among a child with TB meningitis who had recurrent seizures due to meningitis [28], and a woman who developed TB during the first trimester of pregnancy who had four episodes of post-treatment PR: approximately 1 month (17 days after delivery), 7 months, 3 years and 4.5 years after treatment, respectively [32].

We describe an additional case of post-treatment PR 8 years and 4 months after the end of treatment (see Case Vignette).

## Case vignette

A 40-year-old woman presented with a new enlargement of a cervical lymph node still residual eight years and four months after having been treated for drug-sensitive cervical lymph node tuberculosis in another hospital. She had minimal residual nodes after her treatment, but with waxing and waning thereof throughout those almost nine years. One node had increased in size during the most recent 12 months (approximately 2 × 1cm), which had become painful in the last three months, and led to her seeking care. She had no systemic symptoms. She was originally from a highly TB endemic area in the Mediterranean basin, and had returned to visit family regularly, yet had not had a known contact with a tuberculosis patient. She was HIV-negative and had no other important medical history. A lymph node aspirate was both auramine and PCR positive, with negative genotypic resistance testing for isoniazid and rifampicin. Due to the latency period since her original treatment, a paradoxical reaction was considered less likely; hence, the decision to retreat for lymph node tuberculosis was made without awaiting the culture result. She was re-treated for six months, with minimal change to the lymph node. The culture of the aspirate at presentation remained negative and treatment did not improve her symptoms, the combination of which led to revision of the diagnosis to a late post-treatment paradoxical response The treatment was stopped, after which the symptoms continued to wax and wan. Six months later, she presented with an increase in the size of the same lymph node with a new draining fistula. Material from the fistula was auramine negative, PCR positive, and culture negative. The options of surgical excision versus steroids were discussed, and the patient chose the latter. We initiated her on a low dose of 20mg prednisone daily. After a period of four weeks, this had had little impact on her symptoms, but she experienced substantial side effects of the medication, leading to prednisone discontinuation. Due to the minimal symptoms, we neither championed alternative anti-inflammatory agents, nor excision. She was initially observed for a further six months, after which the lymph node was no longer palpable, and the fistula was dry. Since then, about 1.5 years at the time of writing, she has remained relapse-free.

## Timing of post-treatment PR

We were able to extract the exact timing of 30 out of 112 confirmed post-treatment PR cases and 27 out of 42 possible post-treatment PR cases from two of the trials, eight of the cohort studies and 15 case reports (Tables 1 and 2), and of the case vignette included (Table S3, appendix). Figure 2 shows the distribution of reported timing of post-treatment PR cases, stratified by category.

## Risk factors for post-treatment PR

There were three studies that investigated risk factors for PR after the end of treatment, which were all in the same (expanding) LN TB cohort from South Korea [8, 9, 20]. Park and colleagues identified only younger age at diagnosis as an associated factor [20]. Their analysis was underpowered and not adjusted for confounding factors, and may have been affected by multiple testing. Yu et al. confirmed younger age as a risk factor in their larger cohort. They also reported that post-treatment PR was associated with PR during treatment: the incidence increased from 6% among those without PR during treatment, to 12% among those with early PR (0–4 months) and 23% among those with late PR (after 4 months) during treatment [8]. A prior PR during treatment was reported in 50% of the case reports included (Table 2), but was reported poorly in most of the cohort studies (Table 3).

## Diagnostic approach

There are no studies focused on diagnostic approach to recurrence of symptoms post-treatment. From the studies included, it is evident that some authors went to greater lengths to diagnose or exclude microbiological relapse than others.

As is to be expected, the case reports showed mixed diagnostic approaches (Table 2). Most lymph node locations of PR were (needle) biopsied, but in many cases no culture was done (only histology). In the CNS locations of PR, there was only one description of a biopsy of a suspected brain lesion [23], but the majority had only CT or MRI scans done, sometimes combined with CSF analysis.

The trial on PTB treatment reported performing sputum smear and culture in case of recurrent pulmonary symptoms [13].

The cohort studies and trials on LN-TB reported performing fine-needle aspirations and/or excisional biopsies of the recurrent nodes after treatment, which were investigated both histologically and microbiologically (smear and culture, see Table 4). Polesky et al. described an evolving diagnostic approach: as their expertise grew, they were less likely to intervene [18]. They employed a 1–4 week observational period, after which they biopsied the nodes if they had not regressed by then. This approach was also proposed by other authors [8, 9, 20].

**Table 4:** Proposed approach of recurrent symptoms after the end of treatment, stratified by location of disease

Location	Lymph node		Pulmonary		Central nervous system	
Differential diagnosis	Relapse, PR		Relapse, reinfection, bacterial infection in bronchiectasis, PR		Relapse, PR, other pathology	
Clinical status	Not systemically ill	Systemically ill	Not systemically ill	Systemically ill	Tuberculoma	Meningitis (with or without tuberculoma)
Diagnostic approach	4 week observational period, thereafter (ultrasound-guided^a^) FNA/biopsy + TB culture	Immediate (ultrasound-guided^a^) FNA/biopsy + TB culture	Chest X-ray Sputum smear/PCR^b^ + TB culture + normal culture	Chest X-ray Sputum smear/PCR^b^ + TB culture + normal culture	CT or MRI brain If possible: biopsy + TB culture	CT or MRI brain CSF cells/chemistry + PCR + TB culture If possible: biopsy + TB culture
Treatment approach	Options of No treatment Corticosteroids if TB relapse excluded Repeated aspiration Excision (if multiple recurrences)	Corticosteroids if TB relapse excluded	If PCR + : infection prevention measures If bronchiectasis: consider antibiotics Await TB culture results	If PCR + : infection prevention measures If bronchiectasis: antibiotics If smear positive, consider TB retreatment	Corticosteroids Await TB culture results TB retreatment may be considered if biopsy not possible	Corticosteroids Start TB retreatment while awaiting TB culture results

*FNA* fine needle aspiration, *PCR* polymerase chain reaction, *PR* paradoxical reaction, *TB* tuberculosis

^a^Depending on local practice and availability

^b^PCR can stay positive up to 2 years after the end of treatment [48], smear is more likely to be useful in diagnosing relapse

## Management approach and outcome

### Re-treatment

The cohort studies that included substantial follow-up after treatment described their management approach of post-treatment PR; the other cohort studies, which mainly consisted of cohorts on PR during treatment of which a minority were post-treatment cases did not report this separately [4, 7, 17, 19, 21, 22]. The majority described a wait and see approach; two cohorts (Yu et al. and Polesky et al.) described a change in practice that occurred during the course of their study: “As our experience in treating patients with this disease entity [recurrent adenopathy] grew, our inclination to consider retreatment in the absence of a positive culture diminished.” [18].

Of the included case reports, five out of 15 cases were reported as having been re-treated; four of which in the context of CNS disease (Table 2). They reported good clinical response in terms of becoming asymptomatic, although one author (Krishnaraj et al.) reported treating a subsequent relapse of the PR with steroids without TB treatment with the same response [24]. Armange et al. described re-treatment of a third recurrence of LN PR at the expressed wish of the patient, which had no effect [32].

The case descriptions from the cohort studies only included details on management of ten cases; six of which were retreated (three of those despite negative cultures). Three were re-treated in the context of pulmonary or pleural possible PR (two of which had negative sputum cultures).

### Corticosteroids

Despite corticosteroids being the mainstay of PR treatment, their use was not very frequently described. One of the South Korean cohorts reported use in 3% of the patients with post-treatment PR [20]. Yu et al. reported corticosteroid use in 15% of their late PR patients, of whom 80% were still during treatment (this proportion was not reported among the post-treatment PR patients separately). Their earlier cohort reported no corticosteroid use in post-treatment PR, in contrast to 56% among those with PR during therapy [9]. Of the included case-reports, only three reported steroid use; one of those was among the five retreated cases.

### Interventions

Other treatment modalities reported were (repeated) aspiration and excision, both of which were reported with similar frequency as steroid treatment. Polesky et al. reported the patients who required multiple interventions (aspiration or excision) were all immunosuppressed or paediatric patients [18]. The risk of development of a chronic fistula after aspiration was low.

### Outcome

The majority of the included cases did not receive any form of treatment or management, and resolved spontaneously. The outcome of patients was generally favourable. Two deaths were reported, and both were complications unrelated to TB itself: one related to myocardial infarction post-small bowel resection for PR-related intestinal perforation [33], and one fatal bronchopneumonia (TB culture negative) with *E. coli* septicaemia and acute liver failure [13].

## Pathophysiology of late PR

Our graphic overview of the timing of incident PR after the end of treatment suggests a clustering linked to the end of treatment, followed by a lower incidence over a long period of time (Fig. 2). Park et al. also collected timing of PR during treatment and found a bimodal peak in the incidence of PR: one early during treatment, followed by one around the end of treatment [20]. The latter is similar to the peak we show, although ours is lower as we excluded PR cases that occurred at the end of treatment [36–40]. It is unknown whether the peaks early and late during treatment represent different immunological phenomena, or are in fact the same but somehow triggered only towards the end of treatment.

Several authors have speculated on the underlying pathophysiology of late-occurring paradoxical reactions. They are hypothesized to be immunological responses to *Mycobacterium tuberculosis* antigen; either the immune responses were blunted at presentation due to immune suppression (either due to TB disease itself, or to other factors such as HIV) and were restored due to TB treatment or ART, or the immune responses were prolonged because of a very large *Mycobacterium tuberculosis* antigen load at presentation. Late reactions in the latter situation could be attributed to delayed release or inefficient clearance of the antigen, for example due to lower drug penetration (e.g., in CNS disease, as suggested by Cheng et al. and Shah et al. [6, 28]). The former explanation fits very well with the multiple case reports of recurrent episodes of post-treatment PR during immune reconstitution in HIV-positive patients.

Further hypotheses regarding post-treatment PRs are that there could be genetic factors that result in poor clearance of Mtb antigen by macrophages, resulting in large amounts of residual antigen which drives PR, and whether some of these PRs could actually be the result of a low level of microbiological relapse in a lymph node that is then contained by the immune response giving rise to node enlargement and subsequent resolution and negative cultures. This latter hypothesis could fit with our increasing understanding about incipient and subclinical pulmonary TB and how it may run a waxing-and-waning course [41, 42].

There were substantial differences in incidence of post-treatment PR between cohorts, for which there could be various explanations. There were greatly varying lengths of follow-up, with the studies with longer follow-up finding higher incidences of post-treatment PR. There was a lack of studies from sub-Saharan Africa, which could be explained by the fact these patients are simply retreated given TB burden and health system factors. Differences could also be due to different host genetic background. There are reports that suggest inflammatory responses in TB and after initiation of TB treatment differ by ethnic background [43], which could be part of the explanation behind geographic variation in reports of PR incidence [7]: people originating from South Asia were more likely to have LN PR and less systemic symptoms whereas people originating from sub-Saharan Africa were more likely to have pulmonary PR. A study of the role of *Mycobacterium tuberculosis* strain in PR showed no association [44].

Interestingly, the majority of the references we identified from older literature were from the pre-HIV era and did not refer to the term ‘paradoxical reaction’. The publication of the first trial on treatment of lymph node TB from 1979 states: “It is possible that such enlargement and appearance of new nodes or re-appearance of nodes which had previously subsided may be related to hypersensitivity to tuberculoprotein released at intervals from disrupted macrophages”. An opinion piece from 1990 summarising the evidence of several clinical trials states the following: “After chemotherapy nodes can enlarge or appear afresh, usually transiently. Such events do not imply relapse, nor does the persistence of nodes presage relapse.” [45]. Somehow it seems that in the years following, this knowledge was lost, with the majority of the publications between 2000–2015 suggesting extension of treatment in the case of residual or new nodes during treatment [46], or retreatment upon the appearance of new nodes after the end of treatment.

## Proposed approach

We propose that the management of post-treatment PR should be differentiated by location of disease; these are summarized in Table 4. The majority of the literature was on LN TB. From the literature we reviewed, the prognosis of LN post-treatment PR is favourable, and its likelihood is higher than microbiologic relapse. Therefore, it seems safe to include a 4-week observational period before undergoing additional diagnostic testing to exclude relapsed TB disease, as suggested by Park et al. [20].

It is important to note here that none of the studies included were from very high transmission settings such as southern Africa, where up to 30% of annual TB notifications are in patients who have been previously treated for TB [47]. It is unclear how many of these could be in fact post-treatment PRs (or secondary bacterial infections in the context of post-TB bronchiectasis) rather than recurrences, but our data suggest this percentage would be low except in the case of LN-TB patients.

The cornerstone of diagnosis of post-treatment PR is negative mycobacterial culture, as PCR can be false-positive due to the persistence of non-viable mycobacteria after successful treatment [48]. However, there are some concerns that culture is insufficiently sensitive to exclude microbiological relapse [20]. As always, clinical judgement is essential to aid in this assessment. FDG PET scans could be helpful in recognizing nodes at risk of relapse [49, 50], but it is unlikely this can help differentiating PR from relapse. A new TB-specific reagent, 2-[18F]fluoro-2-deoxytrehalose ([18F]FDT), has the potential to identify pathogen viability and might allow such differentiation in the future [51].

Despite the good evidence for 6 months treatment duration for lymph node TB [16, 52], there were still authors who proposed extension of treatment in the event of non-resolution of lymph nodes at the end of the regular treatment period [53]. Our data showed that the incidence of post-treatment PR was the same, irrespective of the duration of treatment, thereby supporting that extended treatment is not beneficial.

The evidence summarized here points towards a conservative approach for LN post-treatment PR, provided cultures of LN sample remain negative (if positive, the patient should be retreated). Should the symptoms persist or worsen, the first-choice treatment is corticosteroids with the option to expand to other anti-inflammatory agents for which the evidence is increasing [54, 55]. Two cases of prolonged paradoxical reactions that developed during treatment in HIV-positive individuals were reported which responded favourably to anakinra employed as a steroid-sparing approach [56]. Even in relatively recent papers, the use of excision and/or (repeated) aspiration for LN-PR was described [8], although in the series of BTS trials neither seemed to confer benefit [45]. This approach could be considered, as the risk of development of a chronic fistula seems low on the basis of the studies reviewed.

However, in the case of CNS localization, diagnostic procedures and treatment with corticosteroids should not be delayed (Table 4). In the case of pulmonary TB, one should investigate immediately, and also consider secondary infection (Table 4).

## Limitations of the review

Limitations of our narrative review were that for the graphs on timing of post-treatment PR it would have been useful to be able to plot PR during treatment as well. However, this was not feasible due to the large number of studies we would have had to include, and to the low likelihood of being able to extract timing of individual patients; for this, an individual-level data meta-analysis would be required. A further limitation to the description of the timing is that follow-up time of the included cohorts differed substantially. Another important limitation was the lack of studies from African settings, which limited the generalizability of the findings. Furthermore, the review mainly includes lymph node TB and HIV-negative TB patients, which limits the generalizability of our findings to all types of TB. Last, we do not expect the advance of diagnostic techniques to identify *M. tuberculosis* to have affected our findings, as these are not based on viability of the mycobacterium. Culture has remained the mainstay of excluding recurrent disease.

## Conclusion

Post-TB treatment PR occurs quite frequently, particularly among LN-TB patients. Reported incidence of confirmed post-treatment PR in LN-TB was between 3 and 14%, and PR was much more frequent than relapse in this population. Incidence rates were much lower among all TB populations (0–2%). There were no estimates of the incidence among CNS-TB patients, but a number of case reports. We found four reports of pulmonary or pleural TB post-treatment PR cases. Risk factors of post-treatment PR identified were younger age at initial diagnosis and having a PR (later) during treatment.

Post-treatment PR most frequently occurs within the first 6 months after the end of treatment, followed by incident cases that occur over the course of several years after the end of treatment. The latest case reported was 10 years after treatment [23]. The data presented do not suggest a difference in timing of PR between PR locations.

The mainstays of diagnosis and management are negative mycobacterial cultures and anti-inflammatory treatment, respectively. In LN-TB recurrent symptoms, due to the favourable prognosis it is warranted to observe for spontaneous regression for a few weeks. In the case of CNS-TB recurrent symptoms, immediate investigation and anti-inflammatory treatment should be undertaken with the consideration of TB retreatment.

## Supplementary Information

Below is the link to the electronic supplementary material.

Supplementary file1 (DOCX 29 KB)

## Data Availability

All data are publicly available (as published data), and are included in summary format in Tables and Figures. The timing data underlying Figure 2 are included in the appendix.
