# Supplementary material for: Post-tuberculosis treatment paradoxical reactions
Source: Infection. 2024 Jul 2;52(5):2083–95. doi: 10.1007/s15010-024-02310-0 (PMC11499416; doi:10.1007/s15010-024-02310-0)
Supplement: Supplementary file 1 — Supplementary file1 (DOCX 29 KB) [file 15010_2024_2310_MOESM1_ESM.docx]

**Appendix**

**Table S1.** Search strategy in PubMed

| Patient | Patients with a successfully treated episode of tuberculosis, presenting with symptoms of recurrence | (Tuberculosis [MeSH] OR  Tuberculosis, Lymph Node [MeSH]) |
| --- | --- | --- |
| Intervention |  |  |
| Comparison |  |  |
| Outcome |  | AND  Paradox*  AND  (Recurrence [MeSH] OR  Paradox* OR  (Residual lymph nodes) OR  Relapse OR Reinfection OR  Post-treatment OR  Late onset) |

**Table S2.** Incidence per treatment arm of included trials

The 60M follow-up of the BTS research committee did not provide the treatment allocation of the one additional PR after the 36M follow-up report, hence was excluded from this table.

**Table S3.** Timing of post-treatment paradoxical reactions, ordered by time since the end of treatment and stratified by category of diagnosis. Please see the reference list of the main manuscript for the full references.

| First author | Number | Months since start TB-Rx | Months since end TB-Rx | Category |
| --- | --- | --- | --- | --- |
| Seok | 1 | NR | 2 | possible |
| Shah | 1 | 20 | 2 | possible |
| Worodria | 1 | 9 | 3 | confirmed |
| Worodria | 1 | 9 | 3 | confirmed |
| Choi | 1 | 9 | 3 | possible |
| Ramos | 1 | 12 | 3 | confirmed |
| Kondo | 1 | NR | 3 | confirmed |
| Worodria | 1 | 10 | 4 | possible |
| Takeshima | 1 | 22 | 4 | possible |
| Mert | 1 | 16 | 4 | confirmed |
| Malhotra | 1 | 20 | 4 | possible |
| Worodria | 1 | 11 | 5 | possible |
| Takao | 1 | 12 | 6 | confirmed |
| Krishnaraj | 1 | 12 | 6 | confirmed |
| Prasai | 1 | 24 | 6 | confirmed |
| Armange | 1 | 13 | 7 | confirmed |
| Yalcinsoy | 1 | 20 | 8 | confirmed |
| Shah | 1 | 24 | 11 | possible |
| Worodria | 1 | 19 | 11 | confirmed |
| Lee | 1 | 31 | 19 | possible |
| Carter | 1 | 33 | 22 | confirmed |
| Seok | 1 | NR | 32 | possible |
| BTS 1984 | 1 | 39 | 33 | possible |
| Huyst, van | 1 | 46 | 34 | possible |
| Brown | 1 | 35 | 41 | possible |
| BTS 1984 | 1 | 51 | 45 | possible |
| Hermans | 1 | 106 | 100 | confirmed |
| Machida | 1 | 132 | 120 | confirmed |
| Park 2013 | 3 |  | 2 | confirmed |
| Park 2013 | 2 |  | 3 | confirmed |
| Park 2013 | 1 |  | 3 | possible |
| Park 2013 | 3 |  | 4 | confirmed |
| Park 2013 | 2 |  | 5 | confirmed |
| Park 2013 | 1 |  | 6 | confirmed |
| Park 2013 | 1 |  | 8 | confirmed |
| Park 2013 | 1 |  | 10 | confirmed |
| Park 2013 | 2 |  | 17 | confirmed |
| Park 2013 | 1 |  | 26 | confirmed |
| Park 2013 | 1 |  | 28 | confirmed |
| Park 2013 | 1 |  | 30 | confirmed |
| Park 2013 | 2 | 7 |  | confirmed |
| Park 2013 | 1 | 8 |  | confirmed |
| Park 2013 | 2 | 9 |  | confirmed |
| Park 2013 | 1 | 9 |  | possible |
| Park 2013 | 1 | 10 |  | possible |
| Park 2013 | 5 | 10 |  | confirmed |
| Park 2013 | 1 | 12 |  | confirmed |
| Park 2013 | 2 | 14 |  | confirmed |
| Park 2013 | 1 | 15 |  | confirmed |
| Park 2013 | 1 | 18 |  | confirmed |
| Park 2013 | 1 | 19 |  | confirmed |
| Park 2013 | 1 | 23 |  | confirmed |
| Park 2013 | 1 | 26 |  | confirmed |
| Park 2013 | 1 | 27 |  | confirmed |
| Park 2013 | 2 | 36 |  | confirmed |
| BTS 1985 | 6 |  | 3 | possible |
| BTS 1985 | 4 |  | 6 | possible |
| BTS 1985 | 2 |  | 18 | possible |
| BTS 1985 | 6 | 12 |  | possible |
| BTS 1985 | 1 | 15 |  | possible |
| BTS 1985 | 3 | 24 |  | possible |
| BTS 1985 | 2 | 36 |  | possible |

NB Cases from Park 2013 and BTS 1985 were taken from tables, therefore it could not be ascertained which timing since start of treatment belonged with which timing since end of treatment.
